# Relationship between the characteristic traits of polycystic ovary syndrome and susceptibility genes

**DOI:** 10.1038/s41598-020-66633-2

**Published:** 2020-06-26

**Authors:** So-hyeon Hong, Young Sun Hong, Kyungah Jeong, Hyewon Chung, Hyejin Lee, Yeon-Ah Sung

**Affiliations:** 1 Division of Endocrinology and Metabolism, Department of Internal Medicine, College of Medicine, Korea University, Seoul, South Korea; 2 Division of Endocrinology and Metabolism, Department of Internal Medicine, Ewha Womans University College of Medicine, Seoul, South Korea; 3 Department of Obstetrics and Gynecology, Ewha Womans University College of Medicine, Seoul, South Korea

**Keywords:** Endocrine system and metabolic diseases, Medical research

## Abstract

Polycystic ovary syndrome (PCOS) is a highly complex disorder influenced by genetic and environmental factors. Previous genome-wide association studies (GWAS) on Han Chinese, Korean, and European populations identified multiple PCOS-susceptible loci; however, only a few studies reported the association of susceptibility genes with disease phenotypic traits. This cross-sectional study aimed to investigate the association between PCOS susceptibility genes from GWAS and disease-related clinical features. A total of 1,810 reproductive-aged women were recruited, including 927 control women and 883 women with PCOS, diagnosed based on the European Society for Human Reproduction and Embryology criteria. Genomic DNA was extracted and genotyped, and a Bonferroni test was performed to determine the association between 12 independent SNPs and the clinical features of PCOS. In women with PCOS, rs11031006, nearest to *FSHB*, was significantly associated with free testosterone (*P* = 1.94 × 10^−3^) and luteinizing hormone (*P* = 1.96 × 10^−3^) levels. The menstruation number per year, ovarian follicular number, ovarian volume, and insulin sensitivity index were not associated with any SNP. In the control group, no SNPs were associated with any PCOS traits. Collectively, our results suggest that *FSHB* may play an important role in the development and progression of PCOS.

## Introduction

Polycystic ovary syndrome (PCOS) is a common endocrine disorder affecting 5–10% of reproductive-aged women worldwide^1,2^. PCOS is characterised by not only hyperandrogenism and irregular menstrual cycles, which are diagnostic criteria components for PCOS, but also hyperinsulinemia and metabolic disturbance, which lead to an increased risk of type 2 diabetes, dyslipidaemia, and cardiovascular diseases^2–4^.

PCOS is a multifactorial disease known to be influenced by both genetic and environmental factors. Over 200 genes have been proposed to be associated with PCOS and its clinical features, including *CYP11A1, Follistatin, INSR, CAPN10, FBN3, IDE*, and *HSD17B5*; however, attempts to replicate the identification of these genes in other studies have failed or have not been made^5–11^. Recently, genome-wide association studies (GWAS) reported susceptible PCOS loci in Han Chinese and Caucasian populations. In the Han Chinese study, 11 susceptible genes for PCOS were identified, including *LHCGR*, *FSHR*, *THADA*, *C9orf3*, *DENND1A*, *YAP1*, *RAB5B/SUOX*, *HMGA2*, *TOX3*, *INSR*, and *SUMO1P1*^12,13^. The European study, conducted on a Caucasian population, found two novel genes, *FSHB* and *GATA4/NEIL2*, and one identical locus previously found in the Chinese study, *C9orf3*^14^. We also conducted a GWAS on a Korean population and identified one novel gene, *KHDRBS3*^15^. However, a GWAS is a non-candidate-driven approach; thus, it does not offer direct insights into the pathogenesis or clinical implications of PCOS.

A few studies have reported an association between susceptibility genes identified in GWAS and phenotypic characteristics. A Chinese group reported that an A homozygote at rs13429485 near *THADA* was associated with increased luteinizing hormone (LH) and testosterone levels, a C homozygote for rs12478601 near *THADA* was associated with increased levels of low-density lipoprotein cholesterol, and G-containing rs2479106 minor alleles near *DENND1A* were associated with elevated insulin levels after 75-g oral glucose tolerance tests (OGTTs) in PCOS^16^. Another study demonstrated that A-containing rs11031010 near *FSHB* was associated with higher LH levels in PCOS^17^. A European study found that the G allele in rs705702 near *RAB5B* and *SUOX* was associated with glucose and insulin levels after 75-g OGTT in patients with PCOS^18^.

As PCOS exhibits different characteristics and multiple phenotypes exist, the identification of single nucleotide polymorphisms (SNPs) and the assessment of their correlation with distinctive PCOS phenotypes are important to understand the pathophysiology of PCOS; this may facilitate the development of novel treatment strategies. Thus, this study aimed to investigate the association between the susceptibility genes for PCOS identified via GWAS and the reproductive and metabolic manifestations of the disease.

## Results

The PCOS group consists of 438 of type A (49.6%), 16 of type B (1.8%), 91 of type C (10.3%), and 338 of type D (38.3%). The clinical characteristics of the subjects are shown in Table 1. The women with PCOS were younger (23 years vs. 26 years, *P* < 0.01) and more obese (21.4 kg/m^2^ vs. 20.7 kg/m^2^, *P* < 0.01) than the control group. Additionally, the women with PCOS exhibited higher fasting insulin levels (5.71 mIU/L vs. 1.20 mIU/L, *P* < 0.01) and post-load glucose levels (101 mg/dL vs. 94 mg/dL, *P* < 0.01) and insulin levels (43.7 mIU/L vs. 16.6 mIU/L, *P* < 0.01) as well as lower insulin sensitivity index (ISI) than the controls (0.081 vs. 0.107, *P* < 0.01).

**Table 1:** Basal characteristics of the controls and women with PCOS. Data are presented as the geometric means (95% CI) or n (%).

	PCOS (*n* = 883)	Control (*n* = 927)	*P*
Age (years)*	23 (20, 27)	26 (23, 29)	<0.01
Body mass index (kg/m^2^)*	21.4 (19.6, 23.9)	20.7 (19.3, 22.4)	<0.01
Oligomenorrhea (%)	776 (87.9)	0 (0)	
Free testosterone (ng/dL)	0.76 (0.44, 1.31)	0.36 (0.21, 0.61)	<0.01
Ovarian follicle number	10.0 (7.0, 14.3)	6.0 (4.2, 8.5)	<0.01
Ovarian volume (cm^3^)	8.5 (5.7, 12.8)	4.9 (3.5, 6.8)	<0.01
Luteinizing hormone (IU/L)	11.3 (6.6, 15.6)	—	
Fasting glucose (mg/dL)	85 (76, 95)	85 (78, 92)	0.88
Fasting insulin (mIU/L)	5.71 (1.12, 29.1)	1.20 (0.11, 12.6)	<0.01
2-hour glucose in 75-g OGTT (mg/dL)	101 (79, 129)	94 (78, 114)	<0.01
2-hour insulin in 75-g OGTT (mIU/L)	43.7 (18.0, 106)	16.6 (3.7, 75.2)	<0.01
ISI (μmol/kg•min)•(pmol/L)^−1^	0.081 (0.049, 0.135)	0.107 (0.082, 0.140)	<0.01

*Data are the median (interquartile range).

CI, confidence interval; ISI, insulin sensitivity index; OGTT, oral glucose tolerance test; PCOS, polycystic ovary syndrome.

The genes near the 12 SNPs from the GWAS for PCOS are shown in Table 2. Among the 12 loci known to be related to PCOS, the 4 SNPs near *LHCGR* (rs10176989, OR = 0.78, *P* = 9.68 × 10^−4^), *TOX3* (rs11075466, OR = 1.29, *P* = 1.73 × 10^−4^), *RAB5B* (rs705704, OR = 1.26, *P* = 2.47 × 10^−3^), and *KHDRBS3* (rs10505648, OR = 0.59, *P* = 3.66 × 10^−5^) were significantly associated with PCOS. The other SNPs did not show statistically significant associations.

**Table 2:** Allele frequency and genotype analysis.

Nearest genes	SNP	Chr.	A1/A2	MAF		OR (95% CI)	*P*
				PCOS	Control		
*FSHB*	rs11031006	11	A/G	0.0587	0.0436	1.37 (1.02, 1.83)	3.31 × 10^−2^
*FSHR*	rs2268361	2	G/A	0.510	0.478	1.14 (1.00, 1.29)	4.67 × 10^−2^
*LHCGR*	rs10176989	2	C/A	0.218	0.264	0.78 (0.67, 0.90)	9.68 × 10^−4^
*TOX3*	rs11075466	16	G/A	0.377	0.319	1.29 (1.13, 1.47)	1.73 × 10^−4^
*RAB5B*	rs705704	12	A/G	0.248	0.207	1.26 (1.09, 1.49)	2.47 × 10^−3^
*KHDRBS3*	rs10505648	8	G/A	0.056	0.090	0.59 (0.46, 0.77)	3.66 × 10^−5^
*YAP1*	rs1894116	11	G/A	0.200	0.168	1.24 (1.46, 2.59)	9.04 × 10^−3^
*THADA*	rs13429458	2	A/C	0.171	0.190	0.88 (0.75, 1.04)	1.26 × 10^−1^
*DENDD1A*	rs2479106	9	G/A	0.252	0.244	1.04 (0.90, 1.22)	5.78 × 10^−1^
*INSR*	rs2059807	19	G/A	0.293	0.292	1.00 (0.87, 1.15)	9.56 × 10^−1^
*C9orf3*	rs4385527	9	A/G	0.202	0.214	0.93 (0.80, 1.09)	3.93 × 10^−1^
*SUMO1P1*	rs6013809	20	A/C	0.382	0.351	1.15 (1.00, 1.30)	4.23 × 10^−2^

A1, minor allele; A2, major allele; Chr., chromosome; CI, confidence interval; MAF, minor allele frequency; OR, odds ratio; PCOS, polycystic ovary syndrome; SNP, single nucleotide polymorphism.

Free testosterone levels, according to the genotypes in the PCOS group, are presented in Table 3. Among the 12 SNPs, women with AA + AG heterozygotes for rs11031006 had higher free testosterone levels than those with G homozygotes, after adjusting for age and BMI (*P* = 1.94 × 10^−3^). In the control group, none of the SNPs showed significant correlations with the free testosterone levels (Supplementary Table 1).

**Table 3:** Free testosterone levels in relation to genotypes in the PCOS group. Data are presented as the geometric means (95% CI). 9.56 × 10^−1^.

Nearest gene	SNP	Genotype	n	Free testosterone (ng/dL)	*P*
*FSHB*	rs11031006	GG	762	0.75 (−0.33, −0.26)	1.94 × 10^−3^
		AA + AG	100	0.84 (−0.23, −0.04)	
*FSHR*	rs2268361	AA	205	0.72 (−0.29, −0.22)	0.060
		GG + GA	657	0.77 (−0.29, −0.22)	
*LHCGR*	rs10176989	AA	602	0.78 (−0.40, −0.28)	6.90 × 10^−3^
		CC + CA	260	0.71 (−0.028, −0.21)	
*TOX3*	rs11075466	AA	337	0.75 (−0.34, −0.24)	0.50
		GG + GA	525	0.77 (−0.31, −0.23)	
*RAB5B*	rs705704	GG	471	0.77 (−0.30, −0.22)	0.35
		AA + GA	391	0.74 (−0.34, −0.25)	
*KHDRBS3*	rs10505648	AA	767	0.75 (−0.31, −0.24)	0.62
		GG + GA	95	0.80 (−0.35, −0.16)	
*YAP1*	rs1894116	AA	551	0.77 (−0.30, −0.22)	0.19
		GG + GA	311	0.74 (−0.36, −0.25)	
*THADA*	rs13429458	CC	335	0.78 (−0.43, −0.14)	0.12
		AA + AC	518	0.74 (−0.31, −0.24)	
*DENDD1A*	rs2479106	AA	463	0.72 (−0.35, −0.27)	0.020
		GG + GA	399	0.80 (−0.28, −0.19)	
*INSR*	rs2059807	AA	426	0.75 (−0.31, −0.22)	0.51
		GG + GA	436	0.77 (−0.33, −0.24)	
*C9orf3*	rs4385527	GG	554	0.76 (−0.31, −0.23)	0.62
		AA + AG	305	0.77 (−0.34, −0.23)	
*SUMO1P1*	rs6013809	CC	334	0.75 (−0.34, −0.24)	0.47
		AA + AC	520	0.76 (−0.31, −0.23)	

All results were adjusted for age and body mass index.

CI, confidence interval; PCOS, polycystic ovary syndrome; SNP, single nucleotide polymorphism.

The LH levels, according to the genotypes in the PCOS group, are presented in Table 4. Women with AA + AG heterozygotes for rs11031006 showed higher LH levels than those with G homozygotes (*P* = 1.96 × 10^−3^). Differences observed for the other SNPs were not statistically significant.

**Table 4:** Luteinizing hormone levels in relation to genotypes in the PCOS group. Data are presented as the geometric means (95%CI).

Nearest gene	SNP	Genotype	n	Luteinizing hormone (IU/L)	*P*
*FSHB*	rs11031006	GG	453	9.54 (2.20, 2.32)	1.96 × 10^−3^
		AA + AG	67	12.95 (2.37, 2.70)	
*FSHR*	rs2268361	AA	124	9.6 (2.13, 2.37)	0.41
		GG + GA	396	10.0 (2.24, 2.38)	
*LHCGR*	rs10176989	AA	373	9.80 (2.21, 2.35)	0.45
		CC + CA	147	10.2 (2.22, 2.44)	
*TOX3*	rs11075466	AA	200	9.44 (2.15, 2.34)	0.20
		GG + GA	320	10.2 (2.25, 2.40)	
*RAB5B*	rs705704	GG	290	9.80 (2.21, 2.36)	0.73
		AA + AG	230	10.1 (2.22, 2.40)	
*KHDRBS3*	rs10505648	AA	458	9.74 (2.21, 2.34)	0.051
		GG + GA	62	11.4 (2.28, 2.62)	
*YAP1*	rs1894116	AA	333	9.92 (2.22, 2.37)	0.80
		GG + GA	187	9.94 (2.20, 2.39)	
*THADA*	rs13429458	CC	319	10.4 (2.25, 2.37)	0.12
		AA + AC	196	9.2 (1.83, 2.39)	
*DENDD1A*	rs2479106	AA	261	9.5 (2.16, 2.33)	0.096
		GG + GA	259	10.3 (2.26, 2.43)	
*INSR*	rs2059807	AA	252	10.5 (2.25, 2.42)	0.16
		GG + GA	268	9.41 (2.17, 2.34)	
*C9orf3*	rs4385527	GG	333	10.3 (2.25, 2.40)	0.17
		AA + AG	187	9.29 (2.14, 2.34)	
*SUMO1P1*	rs6013809	CC	193	9.34 (2.24, 2.34)	0.27
		AA + AC	321	9.70 (2.19, 2.34)	

All results were adjusted for age and body mass index.

CI, confidence interval; PCOS, polycystic ovary syndrome; SNP, single nucleotide polymorphism.

The number of menstrual cycles per year, according to the genotypes in the PCOS group, is presented in Supplementary Table 2. No significant association was observed between the SNPs and the menstruation number per year.

The polycystic ovarian follicular number, according to the genotypes in the PCOS group, is presented in Supplementary Table 3. None of the SNPs showed a statistically significant relationship with the number of ovarian follicles. Similar results were obtained in the control group (Supplementary Table 4).

The ovarian volumes, according to the genotypes in PCOS group, are presented in Supplementary Table 5. None of the SNPs showed a statistically significant relationship with the ovarian volume. Similar results were obtained in the control group (Supplementary Table 6).

The ISIs, according to the genotypes in the PCOS group, are presented in Supplementary Table 7. None of the SNPs showed a statistically significant relationship with the ISI in the PCOS or control groups (Supplementary Table 8). The homeostatic model assessment for insulin resistance (HOMA-IR) showed no significant differences between the PCOS and control groups (Supplementary Tables 9, 10).

## Discussion

In this study, we found that, among 12 susceptibility genes for PCOS, *FSHB* was associated with free testosterone and LH levels, after adjusting for age and BMI, in PCOS women. In contrast, no gene was associated with any clinical features of PCOS in the control group. Thus, these findings suggest that *FSHB* plays an important role in the pathogenesis of PCOS.

Although this was not a replicate study to identify susceptibility alleles in PCOS, we found that *LHCGR*, *TOX3*, *RAB5B*, and *KHDRBS3* were related to PCOS in the study population. Among these genes, *KHDRBS3* has been previously suggested as a candidate PCOS-associated gene in a Korean study population^15^; moreover, *LHCGR*, *RAB5B*, and *TOX3* were reported in a Chinese population^12,13^ but not in a European population^14^, suggesting that ethnicity is an important factor in determining PCOS-susceptible loci. Notably, rs11031006, near *FSHB*, which was associated with free testosterone and LH levels in this study, was not statistically related to PCOS. This conflicting result might be due to the small sample size or the different impacts of clinical characteristics on patients with PCOS and control patients.

Our results showed that rs11031006, near *FSHB*, was associated with multiple PCOS phenotypes, including free testosterone and LH levels. Previous GWAS data showed that *FSHB* was related to PCOS and *FSHB* variants were associated with LH levels, but no association with testosterone levels was found^14,17^. *FSHB* encodes the beta subunit of the follicle stimulating hormone (FSH)^27^. FSH binds to the follicle stimulating hormone receptor (FSHR), which is expressed in the ovarian granulosa cell, and induces oestradiol production and follicle development in the early stages of ovulation^28^. In PCOS, relatively decreased FSH and elevated LH levels stimulate androgen production in theca cells. However, the aromatisation of testosterone to oestradiol is lacking in granulosa cells, which results in hyperandrogenaemia^29^. Higher testosterone levels result in a vicious cycle of negative feedback at the hypothalamic-pituitary axis, decreased sensitivity of oestradiol and progesterone, increased GnRH pulse frequency, and enhanced LH release^30^. Collectively, *FSHB* variants are closely associated with hyperandrogenaemia and higher LH levels in women with PCOS.

Interestingly, we could not find any SNP associated with insulin-related indices, including ISI and HOMA-IR. Insulin resistance is a major pathophysiology of PCOS. Hyperinsulinemia by insulin resistance provokes (1) increased LH secretion, causing ovarian theca cells to release androgens, and (2) pre-antral follicle development arrest resulting in anovulation^31^. Therefore, hyperandrogenism, insulin resistance, and the FSH/LH axis are closely related to each other, exacerbating PCOS pathogenesis and related clinical features. Nevertheless, we could not find any association between any SNP and ISI and HOMA-IR in both PCOS and control groups. The lack of association may be attributed to our study population being composed of young and having normal body weight women who are unlikely to possess advanced metabolic manifestations of PCOS.

Previous studies on European and Han Chinese women showed that *FSHR* variants were associated with PCOS^12–14^. However, we could not find an association between rs2268361 near *FSHR* and any clinical feature of PCOS—free testosterone level, LH level, menstruation number per year, ovarian follicular number and volume, and ISI. These results were similar in women with PCOS and control women. Compared with two other studies, in which PCOS was diagnosed based on the National Institute of Child Health and Human Disease criteria, our study population was recruited according to the European Society for Human Reproduction and Embryology (ESHRE) criteria and included less severe PCOS phenotypes. In the European study, the association between *FSHR* variants and FSH and LH levels was observed in the Greek cohort but not in the Boston cohort^18^.

rs10505648 near *KHDRBS3* is a unique locus for PCOS identified in Korean women^15^. It is a member of the STAR (signal transduction and the activation of RNA) family and regulates gene expression by RNA splicing^32^. It is known to be associated with telomerase activity; shorter telomere length or dysregulated telomerase activity is known to be associated with PCOS^33^ as well as infertility^34^, ovarian insufficiency^35^, and recurrent pregnancy loss^36^. Although no significant difference (*P* = 0.051) was observed between the LH levels in AA homozygotes (9.74 IU/L) and GG + GA heterozygotes (11.4 IU/L) in the PCOS group, the LH level is closely related to PCOS pathogenesis, as previously mentioned. Thus, further analysis, including the in-depth bioactivity of *KHDRBS3* in PCOS and its relevance in different ethnic populations, could assist in elucidating the underlying mechanism.

We could not find any association between rs10176989 near *LHCGR*, rs705702 near *RAB5B*, rs1894116 near *YAP1*, rs13429458 near *THADA*, or rs2479106 near *DENND1A* and the clinical traits of PCOS in both the PCOS and control groups. LHCGR is a G protein-coupled receptor that binds LH, which plays a pivotal role in PCOS pathogenesis^29^. A European study reported that elevated *LHCGR* expression enhanced androgen synthesis in theca cells in patients with PCOS^37^. A Chinese study did not find any association between *LHCGR* and PCOS traits^17^. RAB5B is a member of the RAS oncogene family and is involved in vesicular trafficking at the plasma membrane^38^. A study reported that microRNA-320-targeting of *RAB5B* was significantly attenuated in the PCOS group, compared to a control group, in human follicular fluid^39^. A European study reported that *RAB5B* variants influenced insulin and glucose levels after 12 min of 75-g OGTT^18^. YAP1 is a transcriptional co-activator in the Hippo pathway, which regulates cell proliferation and apoptosis^40^. It is also involved in organ size regulation, including ovarian enlargement and tumorigenesis. Down-regulation of this pathway has been observed in ovarian carcinoma^41^. In ovarian granulosa cells in patient with PCOS, *YAP1* promoter methylation was decreased, with the degree of methylation being dependent on testosterone dose^16^. rs13429458 near *THADA* is associated with PPAR-γ and is involved in apoptosis. A previous genotype-phenotype analysis in China revealed that *THADA* was associated with increased testosterone and LH levels^16^. DENND1A is involved in clathrin-mediated endocytosis, facilitating protein and lipid internalisation, receptor recycling, and membrane trafficking. It is highly expressed in testosterone-producing ovarian theca cells^42^. Increased *DENND1A* expression was identified in PCOS; however, the exact mechanism remains unknown^43^. A previous study revealed that a *DENND1A* SNP in the dominant model resulted in significantly higher insulin levels after 75-g OGTT and an increased risk of insulin resistance in type 2 diabetes patients^44^. Such findings may be attributed to the ethnic composition of the study population, the diagnostic criteria, and the regulatory effects of genetic variation. Further studies are needed to comprehensively evaluate the clinical manifestations of genetic variants in PCOS.

The strength of our study lies in the relatively large and homogeneous study population and the application of strict statistical values for evaluating the association between the 12 susceptibility genes for PCOS and their clinical features. As GWAS reflects ethnic differences, the associations between the susceptibility genes for PCOS and the disease traits identified herein may be characteristic of the Korean population. There are several limitations as well. First, we used dominant model for the analysis due to the small size of the study population containing homozygous minor alleles, so differences among groups having homozygous minor allele, homozygous major allele, and heterozygous allele could not be assessed. Second, we only analysed the candidate SNPs in 12 genes from the previous GWAS; other candidate genes were not analysed. Third, the subjects were limited to relatively young, Asian women with near-normal body weight, so the results might not be applicable to other populations. Fourth, we measured testosterone levels by a chemiluminescent immunoassay, which is generally regarded to be less accurate than techniques involving mass spectrometry. Lastly, we measured LH levels only in the PCOS group; hence, the effect of the genotype of each susceptible SNP on LH levels in the control remains unknown. Further large-scale studies including diverse age and ethnic groups should be conducted to confirm our findings.

In conclusion, we identified that the *FSHB* gene was associated with free testosterone and LH levels in Korean PCOS women but not in control women. This relationship suggests that *FSHB* may play an important role in PCOS development by altering free testosterone and LH levels. Further studies are warranted to validate the results and to explore the underlying pathophysiological mechanisms in the association between FSHB and the development and progression of PCOS.

## Methods

### Subjects

This cross-sectional study was conducted on the study population from a previous GWAS^15^; it consisted of 883 patients with PCOS, diagnosed by the ESHRE criteria, and 927 healthy control women. The ESHRE criteria define PCOS as the presence of any two or more of the following: oligomenorrhea, hyperandrogenism, and polycystic ovary morphology (PCOM)^19^. Oligomenorrhea is defined as less than eight menstrual cycles per year. Hyperandrogenism is defined as levels of total or free testosterone above the 95th percentile based on 1,120 healthy women with regular menstruation cycles, i.e., total testosterone ≥67 ng/dL, free testosterone ≥0.84 ng/dL^20^, or a modified-Ferriman-Gallway (mFG) score ≥3 according to Asian criteria^21^. PCOM is defined as either an ovarian volume >10 cm^3^ or numbers of ovarian follicles with 2–9 mm diameters ≥12. PCOS diagnosis was made after exclusion of other diseases, such as hyperprolactinemia, thyroid disease, congenital adrenal hyperplasia 21-hydroxylase deficiency, androgen-secreting tumour, and Cushing’s syndrome. Cases with a history of hormonal therapy for at least three months were also excluded. Control subjects consisted of women with regular menstruation and without hyperandrogenism or PCOM.

The subjects were recruited from 2008 to 2010 at the Endocrinology and Gynecology Clinics at Ewha Womans University Mokdong Hospital, Seoul, South Korea. The study population included reproductive-aged women who gave informed consent. The institutional review board of Ewha Womans University Medical Center approved the study (IRB No. 187-30) and the study was performed in accordance with the declaration of Helsinki.

### Clinical and laboratory examination

The study participants visited the clinic on the third day of the follicular phase during their menstrual cycle, after an overnight fast. Blood samples were collected from women with amenorrhea on a randomly selected day, and serum progesterone levels were assessed. Women with progesterone levels <4 ng/mL were considered to have anovulation^22^. Subjects underwent a detailed review of their medical history. Anthropometric data (height, weight, and waist circumference) were measured, and body mass index (BMI) was calculated as the weight (kg)/height (m^2^). Hirsutism was scored by a trained nurse using the mFG score (the degree of hair growth from 0 to 4 for each of nine body areas, including the upper lip, chin, chest, upper and lower back, abdomen, upper arms, forearms, thigh, and legs). Venous blood was sampled to detect total testosterone and sex hormone-binding globulin (SHBG) levels using chemiluminescent immunoassays (Siemens, NY, USA, and DPC, Los Angeles, CA, USA), respectively. Free testosterone levels were calculated using Kaufman’s formula (available at http://www.issam.ch/freetesto.htm). The 75-g OGTT was performed, and plasma glucose was measured using the glucose oxidase method (Beckman Model Glucose Analyzer 2, Los Angeles, CA, USA); serum insulin levels were measured via a radioimmunoassay (BioSource, Nivelles, Belgium). The ISI was derived using the following formula: ISI (μmol/kg•min)•(pmol/L)^−1^) = 0.157–4.576 × 10^−5^ × I_120_ - 0.00519 × G_90_ - 0.000299 × I_0_ (I_120_ is the insulin level 120 minutes after 75-g OGTT, G_90_ is the glucose level 90 minutes after 75-g OGTT, and I_0_ is the fasting insulin level)^23^. The HOMA-IR was calculated using the following formula: G_0_ × I_0_/405 (G_0_ is the fasting glucose level)^24^.

An ultrasound examination was performed via 7-MHz transvaginal, transabdominal, or transrectal ultrasonography with a distended bladder in the case of women without a history of intercourse (Logic 400 General Electric, Milwaukee, WI, USA). Ovarian volume was calculated using the simplified formula for an ellipsoid (0.52 × length × width × thickness)^25^.

### SNP determination

Genomic DNA was extracted and genotyped using the HumanOmni 1-Quad array (Illumina, San Diego, CA, USA), as previously mentioned^15^. We selected 12 SNPs in 10 genes from the previous Chinese GWAS study, including 2p 16.3 (rs10176989 near *LHCGR*), 2p16.3 (rs2268361, rs2349415, and rs13405728 near *FSHR*), 2p21 (rs13429458 near *THADA*), 9q22.32 (rs4385527 and rs3802457 near *C9orf3*), 9q33.3 (rs2479106 near *DENND1A*), 11q22.1 (rs1894116 near *YAP1*), 12q13.2 (rs705702 near *RAB5B/SUOX*), 16q12.1 (rs4784165 near *TOX3*), 19p13.3 (rs2059807 near *INSR*), and 20q13.2 (rs6022786 near *SUMO1P1*); one gene from the Korean study, 8q24.2 (rs10505648 near *KHDRBS3*); and one gene from the European study, including 11p14.1 (rs11031006 near *FSHB*)^12–15^. Markers that had a high missing genotype call rate (>1%), high computed average pairwise identity-by-state values (>0.80), low minor allele frequency (MAF < 0.05), and significantly deviated Hardy-Weinberg equilibrium (*P* < 1 × 10^−6^) were excluded^26^.

### Statistical analysis

Statistical evaluations were performed using the SPSS 20.0 software package for Windows (IBM Corp. Chicago, IL, USA). The Kolmogorov-Smirnov statistic was used to analyse continuous variables for normality, and logarithmic transformation was applied to skewed data. Continuous variables were reported as the geometric mean (95% confidence interval) or median (interquartile range) and comparisons between two groups were performed using Student’s *t*-test and *P* values <0.05 were considered statistically significant. The allele frequency and genotype differences were calculated by the PLINK program (http://pngu.mgh.harvard.edu/~purcell/plink/). A dominant model, which test a difference between the group having at least one minor allele and the group having no minor allele, was adopted in the analysis because of the small number of the homozygous minor allele group. For the multiple testing correction for the 12 SNPs examined by the number of clinical features repeatedly, we used a Bonferroni’s correction which *P* values <4.17 × 10^−3^ were considered significant.

## Supplementary information

Supplementary information.
