# Supplementary material for: Relationship between the characteristic traits of polycystic ovary syndrome and susceptibility genes
Source: Sci Rep. 2020 Jun 26;10:10479. doi: 10.1038/s41598-020-66633-2 (PMC7320020; doi:10.1038/s41598-020-66633-2)
Supplement: Supplementary file 1 — Supplementary information. [file 41598_2020_66633_MOESM1_ESM.pdf]

Supplementary Information

Relationship between the characteristic traits of polycystic ovary syndrome and susceptibility genes

So-hyeon Hong, Young Sun Hong, Kyungah Jeong, Hyewon Chung, Hyejin Lee and Yeon-Ah Sung

Supplementary Table 1: Association of free testosterone according to genotypes in control group. Data are presented as geometric mean (95% Confidence interval).

**Nearest gene**	**SNP**	**Genotype**	**n**	**Free testosterone**	***P***
FSHB	rs11031006	GG	737	0.35 (-1.07, -1.00)	0.43
		AA + AG	65	0.38 (-1.11, -0.86)	
FSHR	rs2268361	AA	221	0.37 (-1.07, -0.94)	0.34
		GG + GA	580	0.35 (-1.09, -1.00)	
LHCGR	rs10176989	AA	523	0.35 (-1.09, -1.00)	0.29
		CC + CA	279	0.37 (-1.07, -0.94)	
TOX3	rs11075466	AA	370	0.35 (-1.10, -1.00)	0.56
		GG + GA	432	0.36 (-1.07, -0.97)	
RAB5B	rs705704	GG	497	0.36 (-1.05, -0.96)	0.05
		AA + GA	305	0.34 (-1.14, -1.02)	
KHDRBS3	rs10505648	AA	675	0.35 (-1.09, -0.91)	0.46
		GG + GA	127	0.37 (-1.08, -0.99)	
YAP1	rs1894116	AA	555	0.36 (-1.06, -0.97)	0.13
		GG + GA	246	0.34 (-1.14, -1.01)	
THADA	rs13429458	CC	426	0.35 (-1.10, -1.00)	0.20
		AA + AC	364	0.37 (-1.06, -0.95)	
DENDD1A	rs2479106	AA	439	0.37 (-1.05, -0.95)	0.05
		GG + GA	363	0.35 (-1.12, -1.02)	
INSR	rs2059807	AA	403	0.37 (-1.04, -0.94)	0.016
		GG + GA	399	0.34 (-1.13, -1.03)	
C9orf3	rs4385527	GG	508	0.36 (-1.07, -0.98)	0.84
		AA + AG	294	0.36 (-1.10, -0.98)	
SUMO1P1	rs6013809	CC	331	0.37 (-1.06, -0.95)	0.15
		AA + AC	466	0.35 (-1.10, -1.01)	

All results were adjusted for age and body mass index.

PCOS, Polycystic ovary syndrome; SNP, Single nucleotide polymorphism.

Supplementary Table 2 Association of menstruation number per year according to genotypes in PCOS group. Data are presented as geometric mean (95% Confidence interval).

**Nearest gene**	**SNP**	**Genotype**	**n**	**Menstruation number per year**	***P***
FSHB	rs11031006	GG	774	6.2 (1.81, 1.84)	0.04
		AA + AG	99	5.9 (1.72, 1.82)	
FSHR	rs2268361	AA	204	6.3 (1.81, 1.88)	0.08
		GG + GA	669	6.1 (1.79, 1.83)	
LHCGR	rs10176989	AA	607	6.1 (1.79, 1.83)	0.06
		CC + CA	266	6.3 (1.81, 1.87)	
TOX3	rs11075466	AA	344	6.2 (1.80, 1.85)	0.48
		GG + GA	529	6.1 (1.80, 1.83)	
RAB5B	rs705704	GG	481	6.1 (1.79, 1.83)	0.41
		AA + AG	392	6.2 (1.80, 1.85)	
KHDRBS3	rs10505648	AA	777	6.2 (1.80, 1.84)	0.83
		GG + GA	96	6.1 (1.76, 1.86)	
YAP1	rs1894116	AA	557	6.2 (1.80, 1.85)	0.20
		GG + GA	316	6.1 (1.78, 1.83)	
THADA	rs13429458	CC	523	6.1 (1.79, 1.83)	0.11
		AA + AC	341	6.3 (1.81, 1.86)	
DENDD1A	rs2479106	AA	472	6.3 (1.81, 1.85)	0.11
		GG + GA	401	6.0 (1.78, 1.83)	
INSR	rs2059807	AA	432	6.1 (1.79, 1.83)	0.41
		GG + GA	441	6.2 (1.80, 1.85)	
C9orf3	rs4385527	GG	559	6.2 (1.79, 1.84)	0.69
		AA + AG	314	6.2 (1.79, 1.85)	
SUMO1P1	rs6013809	CC	332	6.1 (1.78, 1.83)	0.23
		AA + AC	533	6.2 (1.81, 1.85)	

All results were adjusted for age and body mass index.

PCOS, Polycystic ovary syndrome; SNP, Single nucleotide polymorphism.

Supplementary Table 3 Association of polycystic ovarian follicular number according to genotypes in PCOS group. Data are presented as geometric mean (95% Confidence interval).

Nearest gene	SNP	Genotype	n	Ovarian follicular number	*P*
FSHB	rs11031006	GG	658	10.1 (2.29, 2.34)	0.08
		AA + AG	76	9.4 (2.16, 2.32)	
FSHR	rs2268361	AA	164	9.8 (2.24, 2.35)	0.63
		GG + GA	570	10.1 (2.28, 2.34)	
LHCGR	rs10176989	AA	521	10.0 (2.27, 2.33)	0.51
		CC + CA	213	10.2 (2.27, 2.37)	
TOX3	rs11075466	AA	289	10.0 (2.27, 2.35)	0.92
		GG + GA	445	10.0 (2.27, 2.34)	
RAB5B	rs705704	GG	398	9.9 (2.26, 2.33)	0.42
		AA + AG	336	10.1 (2.28, 2.35)	
KHDRBS3	rs10505648	AA	80	10.0 (2.28, 2.33)	0.99
		GG + GA	654	10.0 (2.23, 2.38)	
YAP1	rs1894116	AA	474	9.9 (2.26, 2.32)	0.17
		GG + GA	260	10.2 (2.29, 2.37)	
THADA	rs13429458	CC	437	10.0 (2.27, 2.34)	0.66
		AA + AC	289	10.1 (2.27, 2.35)	
DENDD1A	rs2479106	AA	396	10.3 (2.29, 2.36)	0.048
		GG + GA	338	9.7 (2.24, 2.31)	
INSR	rs2059807	AA	369	10.1 (2.27, 2.35)	0.69
		GG + GA	365	10.0 (2.26, 2.34)	
C9orf3	rs4385527	GG	470	10.1 (2.28, 2.35)	0.42
		AA + AG	264	9.8 (2.25, 2.33)	
SUMO1P1	rs6013809	CC	283	10.2 (2.29, 2.37)	0.20
		AA + AC	443	9.9 (2.26, 2.33)	

All results were adjusted for age and body mass index.

PCOS, Polycystic ovary syndrome; SNP, Single nucleotide polymorphism.

Supplementary Table 4 Association of polycystic ovarian follicular number according to genotypes in control group. Data are presented as geometric mean (95% Confidence interval).

Nearest gene	SNP	Genotype	n	Ovarian follicular number	*P*
FSHB	rs11031006	GG	687	6.0 (1.77, 1.82)	0.21
		AA + AG	63	5.7 (1.65, 1.82)	
FSHR	rs2268361	AA	199	5.8 (1.71, 1.81)	0.24
		GG + GA	550	6.0 (1.77, 1.82)	
LHCGR	rs10176989	AA	490	5.9 (1.75, 1.81)	0.37
		CC + CA	259	6.1 (1.76, 1.85)	
TOX3	rs11075466	AA	336	6.0 (1.76, 1.83)	0.48
		GG + GA	414	5.9 (1.74, 1.81)	
RAB5B	rs705704	GG	465	5.9 (1.75, 1.81)	0.40
		AA + AG	285	6.1 (1.76, 1.84)	
KHDRBS3	rs10505648	AA	626	6.0 (1.76, 1.82)	0.40
		GG + GA	124	5.9 (1.70, 1.82)	
YAP1	rs1894116	AA	523	6.0 (1.75, 1.81)	0.71
		GG + GA	226	6.0 (1.75, 1.84)	
THADA	rs13429458	CC	390	6.0 (1.76, 1.83)	0.44
		AA + AC	351	5.9 (1.74, 1.81)	
DENDD1A	rs2479106	AA	416	5.8 (1.73, 1.80)	0.04
		GG + GA	334	6.2 (1.78, 1.85)	
INSR	rs2059807	AA	369	5.9 (1.74, 1.81)	0.24
		GG + GA	381	6.1 (1.77, 1.84)	
C9orf3	rs4385527	GG	464	5.9 (1.75, 1.81)	0.40
		AA + AG	286	6.1 (1.76, 1.84)	
SUMO1P1	rs6013809	CC	311	5.9 (1.74, 1.81)	0.41
		AA + AC	434	6.0 (1.76, 1.83)	

All results were adjusted for age and body mass index.

PCOS, Polycystic ovary syndrome; SNP, Single nucleotide polymorphism.

Supplementary Table 5 Association of polycystic ovarian volume according to genotypes in PCOS group. Data are presented as geometric mean (95% Confidence interval).

Nearest gene	SNP	Genotype	n	Ovarian volume	*P*
FSHB	rs11031006	GG	451	8.7 (2.12, 2.20)	0.02
		GA + AA	60	7.5 (1.92, 2.13.)	
FSHR	rs2268361	AA	132	8.3 (2.04, 2.18)	0.31
		GG + GA	379	8.6 (2.11, 2.20)	
LHCGR	rs10176989	AA	349	8.6 (2.11, 2.19)	0.57
		CC + CA	162	8.4 (2.07, 2.19	
TOX3	rs11075466	AA	181	8.6 (2.10, 2.24)	0.67
		GG + GA	330	8.5 (2.06, 2.18)	
RAB5B	rs705704	GG	266	8.4 (2.08, 2.18)	0.50
		AA + AG	245	8.6 (2.11, 2.21)	
KHDRBS3	rs10505648	AA	448	8.5 (2.11, 2.18)	.0.96
		GG + GA	63	8.6 (2.05, 2.25)	
YAP1	rs1894116	AA	186	8.3 (2.08, 2.16)	0.076
		GG + GA	325	8.9 (2.13, 2.25)	
THADA	rs13429458	CC	294	8.5 (2.10, 2.17)	0.84
		AA + AC	212	8.6 (2.09, 2.38)	
DENDD1A	rs2479106	AA	277	8.5 (2.09, 2.19)	0.93
		GG + GA	234	8.6 (2.09, 2.20)	
INSR	rs2059807	AA	254	8.4 (2.08, 2.18)	0.31
		GG + GA	257	8.7 (2.11, 2.21)	
C9orf3	rs4385527	GG	321	8.5 (2.09, 2.18)	0.64
		AA + AG	190	8.6 (2.10, 2.21)	
SUMO1P1	rs6013809	CC	203	8.5 (2.08, 2.19)	0.54
		AA + AC	303	8.6 (2.11, 2.20)	

All results were adjusted for age and body mass index.

PCOS, Polycystic ovary syndrome; SNP, Single nucleotide polymorphism.

Supplementary Table 6 Association of polycystic ovarian volume according to genotypes in control group. Data are presented as geometric mean (95% Confidence interval).

**Nearest gene**	**SNP**	**Genotype**	**n**	**Ovarian volume**	***P***
FSHB	rs11031006	GG	543	4.93 (1.57, 1.62)	0.04
		GA + AA	46	4.42 (1.39, 1.59)	
FSHR	rs2268361	AA	434	4.87 (1.55, 1.61)	0.60
		GG + GA	155	4.94 (1.55, 1.65)	
LHCGR	rs10176989	AA	381	4.84 (1.54, 1.61)	0.43
		CC + CA	208	4.96 (1.56, 1.65)	
TOX3	rs11075466	AA	267	4.92 (1.55, 1.63)	0.67
		GG + GA	322	4.85 (1.54, 1.62)	
RAB5B	rs705704	GG	369	4.75 (1.53, 1.59)	0.01
		AA + AG	220	5.11 (1.59, 1.68)	
KHDRBS3	rs10505648	AA	496	4.89 (1.56, 1.62)	0.96
		GG + GA	93	4.86 (1.62, 1.65)	
YAP1	rs1894116	AA	407	4.89 (1.55, 1.62)	0.93
		GG + GA	181	4.88 (1.54, 1.64)	
THADA	rs13429458	CC	313	4.89 (1.55, 1.62)	0.92
		AA + AC	268	4.88 (1.54, 1.62)	
DENDD1A	rs2479106	AA	322	4.92 (1.56, 1.63)	0.53
		GG + GA	267	4.84 (1.54, 1.62)	
INSR	rs2059807	AA	290	4.86 (1.54, 1.62)	0.74
		GG + GA	299	4.91 (1.55, 1.63)	
C9orf3	rs4385527	GG	365	4.88 (1.55, 1.62)	0.88
		AA + AG	224	4.89 (1.55, 1.63)	
SUMO1P1	rs6013809	CC	243	4.79 (1.52, 1.61)	0.18
		AA + AC	343	4.97 (1.57, 1.64)	

All results were adjusted for age and body mass index.

PCOS, Polycystic ovary syndrome; SNP, Single nucleotide polymorphism.

Supplementary Table 7 Association of insulin sensitivity index according to genotypes in PCOS group. Data are presented as geometric mean (95% Confidence interval).

Nearest gene	SNP	Genotype	n	ISI	*P*
FSHB	rs11031006	GG	423	0.0809 (-2.55, -2.47)	0.94
		AA + AG	65	0.0832 (-2.63, -2.40)	
FSHR	rs2268361	AA	116	0.087 (-2.54, -2.37)	0.15
		GG + GA	372	0.080 (-2.57, -2.48)	
LHCGR	rs10176989	AA	358	0.082 (-2.56, -2.46)	0.86
		CC + CA	130	0.080 (-2.60, -2.44)	
TOX3	rs11075466	AA	186	0.082 (-2.57, -2.44)	0.79
		GG + GA	302	0.081 (-2.57, -2.46)	
RAB5B	rs705704	GG	213	0.079 (-2.59, -2.48)	0.13
		AA + AG	275	0.084 (-2.54, -2.41)	
KHDRBS3	rs10505648	AA	433	0.081 (-2.56, -2.48)	0.30
		GG + GA	55	0.086 (-2.57, -2.33)	
YAP1	rs1894116	AA	311	0.079 (-2.58, -2.48)	0.26
		GG + GA	177	0.085 (-2.55, -2.24)	
THADA	rs13429458	CC	176	0.081 (-2.68, -2.47)	0.43
		AA + AC	306	0.082 (-2.68, -2.28)	
DENDD1A	rs2479106	AA	240	0.081 (-2.58, -2.46)	0.65
		GG + GA	248	0.082 (-2.56, -2.44)	
INSR	rs2059807	AA	238	0.084 (-2.56, -2.44)	0.55
		GG + GA	250	0.079 (-0.26, -2.44)	
C9orf3	rs4385527	GG	316	0.080 (-2.58, -2.48)	0.20
		AA + AG	172	0.083 (-2.54, -2.41))	
SUMO1P1	rs6013809	CC	185	0.081 (-2.58, -2.45)	0.83
		AA + AC	297	0.082 (-2.56, -2.45)	

All results were adjusted for age and body mass index.

ISI, Insulin sensitivity index; PCOS, Polycystic ovary syndrome; SNP, Single nucleotide polymorphism.

Supplementary Table 8 Association of insulin sensitivity index according to genotypes in control group. Data are presented as geometric mean (95% Confidence interval).

**Nearest gene**	**SNP**	**Genotype**	**n**	**ISI**	***P***
FSHB	rs11031006	GG	277	0.107 (-2.26, -2.20)	0.70
		AA + AG	25	0.103 (-2.35, -2.15)	
FSHR	rs2268361	AA	221	0.108 (-2.26, -2.20)	0.68
		GG + GA	81	0.105 (-2.30, -2.19)	
LHCGR	rs10176989	AA	201	0.108 (-2.26, -2.19)	0.41
		CC + CA	101	0.105 (-2.30, -2.20)	
TOX3	rs11075466	AA	138	0.103 (-2.24, -2.16)	0.02
		GG + GA	164	0.110 (-2.31, -2.23)	
RAB5B	rs705704	GG	178	0.105 (-2.24, -2.16)	0.05
		AA + AG	124	0.110 (-2.30, -2.22)	
KHDRBS3	rs10505648	AA	255	0.107 (-2.27, -2.20)	0.95
		GG + GA	47	0.108 (-2.30, -2.16)	
YAP1	rs1894116	AA	204	0.106 (-2.26, -2.19)	0.30
		GG + GA	97	0.108 (-2.31, -2.21)	
THADA	rs13429458	CC	166	0.106 (-2.28, -2.20)	0.62
		AA + AC	134	0.109 (-2.27, -2.18)	
DENDD1A	rs2479106	AA	164	0.109 (-2.25, -2.18)	0.17
		GG + GA	138	0.105 (-2.30, -2.21)	
INSR	rs2059807	AA	143	0.108 (-2.26, -2.18)	0.32
		GG + GA	159	0.106 (-2.29, -2.21)	
C9orf3	rs4385527	GG	186	0.108 (-2.27, -2.20)	0.97
		AA + AG	116	0.106 (-2.28, -2.19)	
SUMO1P1	rs6013809	CC	128	0.108 (-2.27, -2.18)	0.62
		AA + AC	173	0.107 (-2.28, -2.20)	

All results were adjusted for age and body mass index.

ISI, Insulin sensitivity index; PCOS, Polycystic ovary syndrome; SNP, Single nucleotide polymorphism.

Supplementary Table 9 Association of homeostatic model assessment for insulin resistance according to genotypes in PCOS group. Data are presented as geometric mean (95% Confidence interval).

Nearest gene	SNP	Genotype	n	HOMA-IR	*P*
FSHB	rs11031006	GG	450	1.21 (0.04, 0.35)	0.67
		AA + AG	67	1.22 (-0.18, 0.58)	
FSHR	rs2268361	AA	122	1.06 (-0.24, 0.35)	0.37
		GG + GA	395	1.27 (0.07-0.40)	
LHCGR	rs10176989	AA	381	1.30 (-0.33, 0.34)	0.074
		CC + CA	136	1.04 (0.11, 0.42)	
TOX3	rs11075466	AA	200	1.24 (-0.004, 0.44)	0.83
		GG + GA	317	1.20 (-0.006, 0.37)	
RAB5B	rs705704	GG	292	1.18 (-0.03, 0.36)	0.69
		AA + AG	225	1.26 (0.03, 0.43)	
KHDRBS3	rs10505648	AA	453	1.20 (0.03, 0.34)	0.80
		GG + GA	64	1.30 (-0.09, 0.61)	
YAP1	rs1894116	AA	333	1.28 (0.08, 0.41)	0.31
		GG + GA	184	1.10 (-0.17, 0.36)	
THADA	rs13429458	CC	322	1.26 (0.06, 0.39)	0.31
		AA + CA	188	1.15 (0.06, 0.40)	
DENDD1A	rs2479106	AA	257	1.17 (-0.05, 0.37)	0.75
		GG + GA	260	1.26 (0.04, 0.42)	
INSR	rs2059807	AA	256	1.30 (0.08, 0.45)	0.15
		GG + GA	261	1.14 (-0.09, 0.34)	
C9orf3	rs4385527	GG	333	1.24 (0.05, 0.38)	0.45
		AA + AG	184	1.16 (-0.11, 0.42)	
SUMO1P1	rs6013809	CC	201	1.34 (0.011, 0.48)	0.37
		AA + AC	310	1.13 (-0.08, 0.33)	

All results were adjusted for age and body mass index.

HOMA-IR, Homeostatic model assessment for insulin resistance; PCOS, Polycystic ovary syndrome; SNP, Single nucleotide polymorphism.

Supplementary Table 10 Association of homeostatic model assessment for insulin resistance according to genotypes in control group. Data are presented as geometric mean (95% Confidence interval).

Nearest gene	SNP	Genotype	n	HOMA-IR	*P*
FSHB	rs11031006	GG	258	0.25 (-2.02, -0.25)	0.77
		AA + AG	26	0.32 (-1.69, -1.11)	
FSHR	rs2268361	AA	78	0.25 (-1.93, -0.83)	0.80
		GG + GA	206	0.25 (-1.69, -1.05)	
LHCGR	rs10176989	AA	189	0.24 (-1.75, -1.09)	0.70
		CC + CA	95	0.28 (-1.78, -0.79)	
TOX3	rs11075466	AA	129	0.30 (-1.61, -0.79)	0.21
		GG + GA	155	0.22 (-1.89, -1.15)	
RAB5B	rs705704	GG	169	0.33 (-1.42, -0.76)	0.0067
		AA + AG	115	0.17 (-2.27, -1.32)	
KHDRBS3	rs10505648	AA	241	0.22 (-1.81, -1.19)	0.043
		GG + GA	43	0.50 (-1.21, -0.15)	
YAP1	rs1894116	AA	192	0.26 (-1.67, -1.02)	0.92
		GG + GA	91	0.24 (-1.97, -0.93)	
THADA	rs13429458	CC	154	0.21 (-1.98, -1.16)	0.087
		AA + CA	128	0.31 (-1.53, -0.81)	
DENDD1A	rs2479106	AA	153	0.26 (-1.71, -0.97)	0.81
		GG + GA	131	0.24 (-1.83, -1.00)	
INSR	rs2059807	AA	137	0.27 (-1.72, -0.93)	0.65
		GG + GA	147	0.24 (-1.81, -1.04)	
C9orf3	rs4385527	GG	176	0.25 (-1.74, -1.02)	0.99
		AA + AG	108	0.26 (-1.80, -0.93)	
SUMO1P1	rs6013809	CC	119	0.25 (-1.84, -0.95)	0.59
		AA + AC	164	0.26 (-1.67, -0.98)	

All results were adjusted for age and body mass index.

HOMA-IR, Homeostatic model assessment for insulin resistance; PCOS, Polycystic ovary syndrome; SNP, Single nucleotide polymorphism.
